# Artificial intelligence in reproductive medicine

**DOI:** 10.1530/REP-18-0523

**Published:** 2019-04-10

**Authors:** Renjie Wang, Wei Pan, Lei Jin, Yuehan Li, Yudi Geng, Chun Gao, Gang Chen, Hui Wang, Ding Ma, Shujie Liao

**Affiliations:** 1 Department of Obstetrics and Gynecology, Cancer Biology Research Center, Tongji Hospital, Tongji Medical College of HUST, Wuhan, Hubei, People’s Republic of China; 2 School of Economics and Management, Wuhan University, Wuhan, Hubei, People’s Republic of China

## Abstract

Artificial intelligence (AI) has experienced rapid growth over the past few years, moving from the experimental to the implementation phase in various fields, including medicine. Advances in learning algorithms and theories, the availability of large datasets and improvements in computing power have contributed to breakthroughs in current AI applications. Machine learning (ML), a subset of AI, allows computers to detect patterns from large complex datasets automatically and uses these patterns to make predictions. AI is proving to be increasingly applicable to healthcare, and multiple machine learning techniques have been used to improve the performance of assisted reproductive technology (ART). Despite various challenges, the integration of AI and reproductive medicine is bound to give an essential direction to medical development in the future. In this review, we discuss the basic aspects of AI and machine learning, and we address the applications, potential limitations and challenges of AI. We also highlight the prospects and future directions in the context of reproductive medicine.

## Introduction

The term AI was first coined by John McCarthy at the Dartmouth Summer Research Project on Artificial Intelligence in 1955. AI is defined as the ability of machines to learn and display intelligence, which is in stark contrast to the natural intelligence demonstrated by humans and animals. AI has developed rapidly and gradually penetrated our personal and social life since then. In the recent years, computers, driven by computer power, memory, data storage and large amounts of data, have been handling increasingly complex learning tasks with incredible success (Deo 2015). AI applications used in daily life include speech recognition (Abdel-Hamid *et al.* 2014), face recognition (Taigman *et al.* 2014), game AI (Silver *et al.* 2016), intelligent voice assistant (Strayer *et al.* 2017) and self-driving vehicles (Katrakazas *et al.* 2015). There is no doubt that AI applications will become faster, smarter and more accessible. However, the current range of AI applications is still very narrow. Despite progress, achieving universality is still a considerable challenge (LeCun *et al.* 2015, Esteva *et al.* 2017).

In the medical field, breakthroughs in the recent years have led to a dramatic increase in the volume and complexity of biomedical data generated from individuals, biological experiments, hospitals and environmental factors (Andreu-Perez *et al.* 2015), which brings new opportunities and poses challenges in clinical activities. The explosive increase of available biomedical data has exceeded the capability of doctors to extract all meaningful data to gain insights on complex diseases using conventional statistical methods. This calls for a higher-level analysis method to help physicians effectively analyze the data (Andreu-Perez *et al.* 2015, Deo 2015, Obermeyer & Emanuel 2016). AI learns the potential relationships in a large amount of biological data using complex algorithms and uses these insights to assist clinical activities. It can also gain new medical information from successful clinical cases and clinical guidelines to improve its accuracy. AI can reduce the inevitable errors in diagnosis and treatment in human clinical practice and make real-time predictions on health risk (Patel *et al.* 2009, Lee *et al.* 2013, Neill 2013). The broad spectrum of medicine, radiology, cardiology and oncology have benefited from AI application (Esteva *et al.* 2017, Hosny *et al.* 2018, Johnson *et al.* 2018, Tang *et al.* 2018).

Advances in AI applications are constantly promoted by the increasing amount of data available in reproductive medicine. Despite some potential pitfalls, making decisions for infertility patients based on the analysis of medical data is the optimal clinical approach. To reduce the gap between research and clinical practice, we need to focus on combining ART and AI development. Reproductive experts can determine the best treatment for the individual infertility of patients by incorporating AI and machine learning models (Siristatidis *et al.* 2016), which is a significant advancement in the development of ART. Infertility patients, at a personal level, can be provided with the most appropriate therapy, increasing the successful pregnancy rates and reducing the financial burden. At the social level, unnecessary use of medical resources can be avoided leading to a reduction of health care costs (Senders *et al.* 2018).

**Figure 1:**
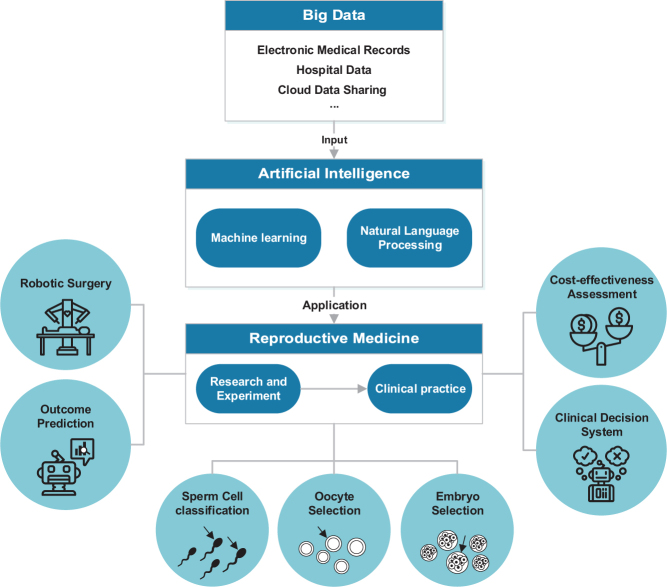
The role of artificial intelligence in Reproductive Medicine. Big data include electronic medical records (EMRs) and other data. EMRs can capture data from various ways and the data is analyzed using AI such as machine learning and natural language processing (NLP). AI has been used in the many aspects of reproduction, from research and experiment to clinical practice. This schematic reviews the seven main applications of AI in reproductive medicine.

Recently, AI is used mainly in the following areas (see Fig. 1): select and predict the sperm cell to improve the success rate of treatment, assess the quality of embryos and oocytes and establish a useful ART prediction model and predict the outcome. Ongoing studies are conducted to define good noninvasive markers to increase the implantation rate and improve the efficacy of ART treatment. An integrated AI component with image analysis would increase recognition efficiency, reduce errors and achieve minimal manual classification workload by providing automatic classification of the sperm, the embryos and the oocytes. However, the main limitations of current studies derive from the quantity and quality of data, which significantly affect the performance, applicability and generalizability of the trained model. In most studies, the data of the models are small in number, single in source and retrospective. There is still a lack of large-scale randomized controlled trials to test the external validity of the algorithms and optimize the use of limited research resources. Furthermore, most research is limited to applying algorithms for classification and prediction, but lack integration of the analysis data obtained. Currently, the applications of AI in reproductive medicine are relatively limited and mostly semi-automatic. More research on the personalized diagnosis and treatment, remote medical expert system and automatic AI-assisted reproduction is needed.

Our aim is to present a brief introduction to the basics of AI methods, with particular reference to its applications in reproductive medicine. This review identified the potential limitations and challenges and discusses the prospects and future directions in the context of reproductive medicine.

## Why does reproductive medicine require AI?

The rapid development of ART such as oocyte and embryo cryopreservation, assisted fertilization, preimplantation genetic testing and embryo selection technologies have greatly improved the clinical pregnancy rate in the 40 years since the birth of the first *in vitro* fertilization (IVF) baby even though many problems remain (Niederberger *et al.* 2018). The quality of embryos is the most critical factor for the success of IVF, but there is still a lack in the methods of judging the quality of the eggs, the sperm and the embryos accurately. Embryo selection methods using a single parameter or algorithm have not been identified. Therefore, it is difficult to predict the probability of a successful pregnancy for each patient and to fully understand the cause of each failure. AI-based methods in reproductive medicine may become a solution to current dilemmas. The primary driver for the development of these applications is the desire to improve the treatment and prognosis for infertility patients, using the large quantities of data provided by complex diagnostic and therapeutic modalities. AI can provide greater efficacy and efficiency in clinical activities, thereby optimizing the treatment cycle of ART.

## Overview of the AI in reproductive medicine

**Figure 2:**
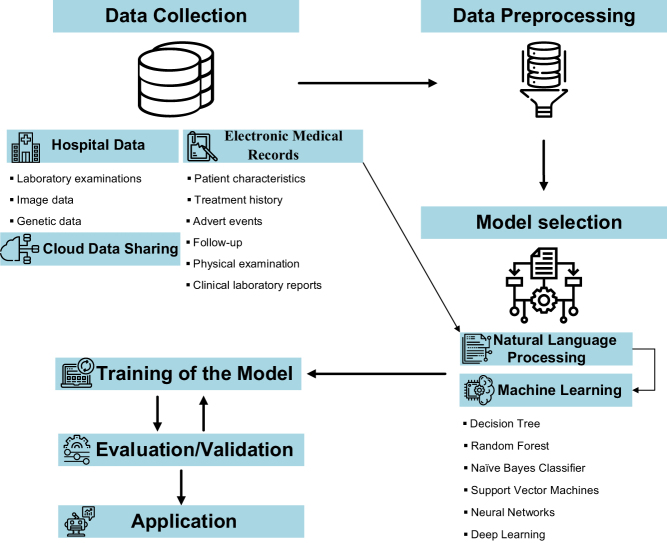
The workflow of artificial intelligence in Reproductive Medicine. This flowchart provides a brief overview of the AI workflow. The first step is the collection of data. The data includes electronic medical records (EMRs), hospital data and cloud data sharing. The second step is data pre-processing. The third step is the selection of the appropriate model. The data is analyzed using artificial intelligence methods such as machine learning and natural language processing (NLP). Then the training dataset is used to train the model. The final steps include the evaluation and validation of the model.

Currently, there are three major categories of AI methods widely used in medical applications: machine learning (ML), natural language processing (NLP) and robotic surgery. The ML method attempts to cluster the features of patients and predict the outcome of diseases by analyzing structured data such as medical imaging and genetic data (Darcy *et al.* 2016). The NLP method extracts and processes meaningful information from unstructured clinical data, such as electronic medical records (EMRs), to complement the structured data (Nadkarni *et al.* 2011, Mehta & Devarakonda 2018). NLP converts the raw data into structured data that the machine can read and analyzes it using ML techniques. For better understanding, the flowchart in Fig. 2 describes the workflow of AI in reproductive medicine.

### Basic knowledge of machine learning

Machine learning (ML) is a subset of AI that enables computer algorithms to model the relationship between a set of observables (input data) with another set of variables (output data) (Camacho *et al.* 2018). The mathematical model allows computers to detect patterns from large complex datasets automatically and uses these patterns to make predictions. Compared to traditional statistics, machine learning focuses on building automated clinical decision systems for the optimal treatment of infertility and predicting pregnancy outcomes to assist doctors in making decisions, rather than merely estimating and scoring disease conditions (Krittanawong *et al.* 2017). To get a more accurate model, machine learning algorithms typically require enormous amounts of high-quality training data. The first step in using machine learning is to collect samples and store them in a form suitable for computational purposes (Bastanlar & Ozuysal 2014). If the input data is insufficient, the final output results are not convincing. Machine learning models were unable to predict the outcome of IVF accurately with an accuracy of 59 and 68%, respectively (Kaufmann *et al.* 1997, Venkat *et al.* 2004). The two studies were methodologically excellent; however, the findings potentially suffered from poor datasets that did not contain strong predictors, such as embryo implantation data. In the era of big data, the rational use of data is both a challenge and an opportunity; machine learning will become an indispensable tool for clinicians.

Machine learning method can be divided into supervised learning, unsupervised learning and reinforcement learning (Jordan & Mitchell 2015). Currently, supervised learning is widely used in almost all AI applications in reproductive medicine. This algorithm uses labeled training data to develop models that can predict a known output. In clinical practice, it requires a dataset with features and labeled outcomes; however, the main drawback is that it requires data that must be labeled by humans, which is time consuming. Unsupervised learning focuses on the hidden structures and relationships in a dataset and requires only the input features in the training data. Since the output labels are not necessary, it can be used to predict unknown results. Reinforcement learning focuses on continuously improving the accuracy of the algorithm through trial and error. It consists of agents that interact with a specific context, wherein the agent uses the reward feedback in determining appropriate behavior and maximizing the reward (Mnih *et al.* 2015, Krittanawong *et al.* 2017, Senders *et al.* 2018). Currently, reinforcement learning is mainly used for medical image processing (Wang *et al.* 2013), personalized medicine (Tseng *et al.* 2017, Tao *et al.* 2018) and robotic surgery (Baek *et al.* 2018). There are relatively few applications for the reinforcement learning method in reproductive medicine, but the method may prove to be useful in robotic-assisted laparoscopy (Jayakumaran *et al.* 2017). Robotic assistance during reproductive surgery can reduce blood loss, postoperative pain and hospital stay and shorten convalescence. Despite the higher cost and longer operating time, the reproductive results are similar to open or laparoscopic surgery; hence, the method can be a reasonable alternative (Jayakumaran *et al.* 2017).

Machine learning requires a large amount of data and robust computing power. The rapid development of graphical processing units (GPUs) makes it possible to process big data. With the gradual development of hardware and software (algorithms), AI and machine learning can dramatically promote the development of reproductive medicine in the near future. We will give a brief introduction of several algorithms, along with application examples.

#### Supervised learning

Supervised learning methods have been applied successfully to image analysis and prediction of ART. Several algorithms have been used: decision tree, random forest, support vector machines (SVMs) and naïve Bayes classifier. Each algorithm has its advantages and disadvantages (see Table 1). In this section, we discussed in detail these methods and application examples in reproduction.

**Table 1:** A brief overview of different machine learning algorithms.

Algorithm	Advantages	Limitations	Application	Reference
Decision tree	Easy to interpret and understand	Risk of overfitting	Cost-effectiveness assessment of elective oocyte cryopreservation and embryo transfer	Guh* et al.* (2011), Mesen* et al.* (2015), Devjak* et al.* (2016), Carrasco* et al.* (2017), van Loendersloot* et al.* (2017)
	Can be combined with other decision techniques			
	Use a white box model			
Random forest	Correct the problem of overfitting in the decision tree	Need a large amount of maintenance work	Prediction of the outcome of IVF and ICSI	Hafiz* et al.* (2017)
	More accurate than results predicted using an individual model			
Support vector machines (SVMs)	Perform well on nonlinear problems	Difficult to train	Classification of sperm cell	Lee* et al.* (2002), Auger (2010), Goodson* et al.* (2011), Santos Filho* et al.* (2012), Tseng* et al.* (2013), Sahoo and Kumar (2014), Goodson* et al.* (2017), Mirsky* et al.* (2017)
	Less risk of error	Difficult to interpret and understand	Embryo selection	
	Powerful model with accurate prediction			
Naïve Bayes classifier	Fast	Problems occur if the input variables are related. Input variables must be statistically independent	Prediction of the implantation outcome based on embryos	Morales* et al.* (2008), Uyar* et al.* (2015)
	Easy to train			
	Easy to understand			
	Perform well on small training datasets			
Neural Network and Deep learning	Algorithms can be adjusted to accommodate new problems quickly	Require massive datasets to train the model	Construction of a predictive model for the outcome of ART	Kaufmann* et al.* (1997), Venkat* et al.* (2004), Milewski* et al.* (2009), Girela* et al.* (2013), Akinsal* et al.* (2018), Cavalera* et al.* (2018)
	Tolerate noise and missing values in data	Highly demanding hardware (computing power) for training		
	Rapid development and broad prospect	Black box. Difficult to understand and interpret		

##### Decision tree and random forest

**Figure 3:**
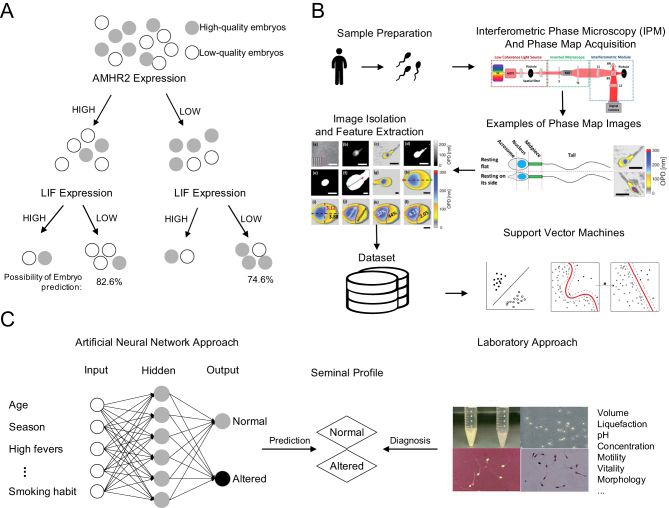
AI applications in reproductive medicine. (A) This decision tree model is conducted to make predictions for the selection of embryos. The model first separates cumulus cells samples upon AMHR2 expression (high or low) and then upon LIF expression (high or low). The gray color represents high-quality embryos and the white color represents low-quality embryos. The combination of high AMHR2 and low LIF expression achieves an 82.6% possibility of predicting a low-quality embryo, and the combination of low AMHR2 and high LIF expression leads to a 74.6% possibility of predicting a high-quality embryo (Devjak *et al.* 2016). (B) The researchers obtained human semen samples from eight healthy donors and acquired the quantitative phase maps of the sperm samples by using the diagram of the optical system. Then they used a program to extract the phase map and features. Finally, the dataset obtained was used to train a two‐class SVM classifier (Mirsky *et al.* 2017). (C) (Girela *et al.* 2013) created an ANN model to produce a decision support system that can help predict the semen parameters based on the data collected by the questionnaires and can support the traditional diagnosis.

Decision tree (DT) and random forest (RF) are robust algorithms that can be used as classification and forecasting tools. DT is a classifier that forms a tree structure (see Fig. 3A), which consists of various nodes, such as root nodes and leaf nodes or decision nodes. The decision nodes carry out several tests to predict the class label and each class is calculated to gain its probability (Sahoo & Kumar 2014). DT is generally the algorithm preferred by physicians and applied in reproductive medicine because it is a white box model. Compared to other algorithms such as neural network, it is easy to interpret and understand. Besides, DT can be combined with other decision techniques to improve the performance of the model. Carrasco *et al.* (2017) developed a hierarchical model based on data mining and used DT to determine optimal embryonic morphokinetic parameters, which can make predictions for the selection of human embryos. The researchers found that the most predictive parameter is the classical morphological score. DT can be used to determine the optimal success rate and cost-effectiveness in pursuing the age of elective human oocyte cryopreservation (Mesen *et al.* 2015), as well as the cost-effectiveness of determining the different embryo transfer strategies in IVF in relation to female age (van Loendersloot *et al.* 2017).

The main advantage of DT is that it can express rules so clinicians can understand and use the algorithm in the dataset efficiently (Sahoo & Kumar 2014). Devjak *et al.* (2016) conducted a model to predict the quality of human embryo based on the cumulus cells gene expression of 17 patients (see Fig. 3A). Two prediction models were tested. The DT model produced easy-to-interpret rules and provided greater informational value in clinical decision making. The major drawback is that it tends to overfit data (Johnson *et al.* 2018). If the model is too complex and suitable for the training data, it is called overfitting. This model fits the training data well with low training errors, but it is unable to predict test data or new samples accurately. Increasing the size of the training set or reducing the complexity of the learning model, such as bootstrapping and bagging, can solve this problem (Domingos 2012, Johnson *et al.* 2018).

RF is a combination of tree predictors such that each tree depends on the values of a random vector sampled independently (Breiman 2001). RF is an ensemble learning method for classification, regression and other tasks. By constructing a multitude of DTs and aggregating the outputs of each weak individual tree, RF corrects the problem of overfitting in the DT (Tin Kam 1995, Breiman 2001). RF performs better than the results predicted using an individual model and requires more maintenance work instead. Hafiz *et al.* (2017) used several data mining techniques to predict the implantation outcome of IVF and intracytoplasmic sperm injection (ICSI). They collected and analyzed the data from 486 patients. Compared with other classifiers, RF and recursive partitioning (RPART) have achieved better prediction results (areas under the ROC curve (AUC) – 84.23 and 82.05%, respectively).

##### Naïve Bayes classifier

Naïve Bayes classifier is a simplified classifier based on Bayesian theorem. Bayesian classifiers have proven good accuracy in complex medical problems. This model is transparent and comprehensive for medical practitioners, which have motivated their choice of this field (Morales *et al.* 2008). Naïve Bayes classifier can perform efficiently because each feature probability can be computed independently. Although the training data are limited, the model parameters can be estimated reliably. Therefore, naïve Bayes classifier works well on small training datasets (Bastanlar & Ozuysal 2014). Naïve Bayes classifier can predict the implantation outcome of individual embryos in an IVF cycle by providing decision support based on the number of embryos transferred. Uyar *et al.* (2015) indicated that by using morphological variables of individual embryos, higher accuracy rates could be obtained regarding human implantation prediction. They selected six classifiers to make a prediction. The naïve Bayes model finally yielded the best predictions on embryo-based implantation with an accuracy of 80.4%, a sensitivity of 63.7% and a false-positive rate of 17.6%. They discovered that the proposed model outperformed expert judgment alone.

In many real problem domains, the predictive accuracy of naïve Bayes classifier is degraded because of the irrelevant predictor variables where the information contribution is overlapped or repeated (Morales *et al.* 2008). The redundant variables can significantly reduce the accuracy of Bayesian classifier (Langley & Sage 1994). A feature selection process can efficiently remove these variables to obtain a valid model, other Bayesian classifiers such as selective naïve Bayes (Langley & Sage 1994), semi naïve Bayes (Pazzani 1996), tree augmented naïve Bayes (Friedman *et al.* 1997) and *k*-dependence Bayesian classifier (Sahami 1996) have been developed.

##### Support vector machines

Support vector machines (SVMs) are widely used supervised learning models in the reproductive domain, which can process data for classification and regression analysis. In most clinical cases, data distributions are not linearly separable. SVMs can capture complex nonlinear relationships using the kernel trick and determine a set of optimal hyperplanes that identify the largest possible margin to separate classes, which is no subjectivity. For this reason, SVMs have been used in the classification of the sperm cell and semen analysis to improve the treatment of male factor infertility. In human semen samples, the sperm cells can be classified as normal or abnormal through morphological semen analysis (Auger 2010). Abnormal sperm cells generally have a lower fertilizing potential. Thus, the morphology is considered a clinical tool dedicated for fertility prognosis; it serves as a way of making decisions regarding the options for ART (Lee *et al.* 2002). Goodson *et al.* (2017) used SVMs, combined with a multiclass DT, to classify the human sperm motility patterns. The method performed well with an overall accuracy of 89.9%. Several studies used SVMs for sperm morphology classification. Tseng *et al.* (2013) applied SVMs to design a sperm classification system that relied mainly on one-dimensional features from 160 human sperms and had an accuracy of 87.5%. Mirsky *et al.* (2017) trained an SVM classifier to classify sperm morphology automatically. The initial dataset consists of 1405 sperm cells from eight human donors. These researchers found that the classifier performed well with an AUC of 88.59%, an area under the precision–recall curve of 88.67%, and a precision of over 90%. Figure 3B displays the main workflow of the proposed method.

Furthermore, SVMs can also be used for embryo assessment. The precise evaluation of embryo viability is a crucial factor in the optimization of IVF treatments. Automated image analysis of embryos can add the objectivity of the selection process. The SVMs that perform a classification task by determining a separation rule between two sets of feature values may be an appropriate classifier for embryo assessment. Santos Filho *et al.* (2012) proposed a method for image segmentation, which could upgrade automatically using SVMs classifiers, to provide a semi-automatic grading of human blastocysts. These investigators obtained an accuracy, ranging from 67 to 92% for human embryo development classification, indicating that automated evaluation tools of embryos based on SVMs are feasible and promising and more work in this area is required.

#### Unsupervised learning, neural networks and deep learning

##### Neural networks and deep learning

Neural networks, inspired by neurons in the human brain, are the most commonly used algorithms for image analysis today (Tang *et al.* 2018). The ultimate objective of the overall neural networks is to learn the appropriate representations to arrive at an accurate prediction for new input data (Camacho *et al.* 2018). The neural network model used in predicting the semen parameters is shown in Fig. 3C. The network employed consists of input layers, hidden layers and output layers. In reproductive medicine, neural networks are mainly used to predict the outcome of IVF. The first attempt was in 1997 (Kaufmann *et al.* 1997); an artificial neural network (ANN) was created to accurately predict pregnancy with an accuracy of 59% for human. It was based on maternal age, number of oocytes obtained, number of embryos transferred and whether embryo cryopreservation was performed. Milewski *et al.* (2009) created an ANN model to predict the adverse outcomes of IVF treatment. The study comprised 1007 cycles of infertility treatment of 899 patients. They reached an excellent result to forecast treatment failure with almost a 90% probability rate. Recently, (Cavalera *et al.* 2018) developed a novel classification method. They predicted the developmental ability of mouse gametes using various models. The feed-forward artificial neural network (FANN) finally achieved a high accuracy of 91.03%. The protocol could also be tested on humans, indicating that it might perform well.

Deep learning is a subfield of representation learning (Tang *et al.* 2018). It typically utilizes neural networks with multiple hidden layers, and each layer performs feature construction for the layers before it. Compared with the neural network, deep learning can handle data with complex structures by using more hidden layers (LeCun *et al.* 2015). The algorithms can automatically design features for many tasks and customize them for one or more specific tasks (Ching *et al.* 2018). Deep learning has become the basis for many modern technologies, such as automatic facial recognition in images and speech recognition (Johnson *et al.* 2018). Deep learning is also capable of fast learning in a large number of samples; this is especially suitable for computer vision (Shameer *et al.* 2018). In medical applications, the most common deep learning algorithms included convolutional neural network (CNN), recurrent neural network (RNN) and deep neural network. CNN performs well when dealing with high dimensional data (data with a large number of features), especially in image data processing. The image data are usually high dimensional because each image typically contains thousands of pixels as features (Jiang *et al.* 2017). In the medical domain, the commonly used solution is decreasing the number of dimensions (features) using dimensionality reduction, and processing the lower dimensional features with the ML algorithms. However, the emergence of CNN has greatly contributed to the resolution of these problems.

Currently, the applications of deep learning in the field of reproduction are rather limited (Miotto *et al.* 2018). Despite its nascence, deep learning in the field of reproduction shows great potential and is still developing. Experts in reproductive medicine must know that deep learning is becoming the most advanced method and will promote the applications of AI shortly. The advantage of deep learning is that it is capable of fast learning in a large number of complex samples and performs well in image data processing. During the treatment of infertility, clinicians obtain ample raw data such as patient characteristics, image data and laboratory examinations data. For example, in sperm cell classification, semen analysis and embryo assessment, vast amounts of images require manual sorting to determine optimal results, which is ideal for processing with deep learning. By collecting raw data from the crowd informatics, genetics, bioinformatics and imaging information, a reproductive health database for large-scale populations can be built. By means of a comprehensive application of deep learning and other methods to analyze the data, researches can establish a systematic training program to perform embryo laboratory work. Recently, CNN has successfully carried out disease diagnosis through computational analysis of images. Esteva *et al.* (2017) identified skin cancer from clinical images using a single CNN; the proportions of correctly predicted malignant lesions and benign lesions are both over 91%. This research demonstrates the principal drawback of deep learning: it requires massive datasets to train a model, which is often unattainable in many biological studies. The researcher can only control the input data and some parameters in the model; hence, the model lacks interpretability, which is hard to understand (Ching *et al.* 2018).

While deep learning has produced outstanding results, one of its main criticisms is that it is used as a black box, which means that we know very little about how such results are derived internally. In the clinical domain, it is not enough to simply produce accurate results, given that many studies are connected to medical decision making and patient treatments. However, many papers ignore interpretability of the model without any explanation, despite this ambiguity. It is crucial to change the black box into the white box as good interpretability is critical for clinical applications and is an important element for information streaming between patients and clinicians. The transformation of deep learning is still in the early stages. One of the most widely used methods is visualizing a trained deep learning model such as a deconvolutional network in image input (Zeiler & Fergus 2014). In addition, maximum activation (Choi *et al.* 2016, Nguyen *et al.* 2017), mimic learning (Che *et al.* 2016) and attention mechanisms (Cho *et al.* 2015) are being studied.

##### Unsupervised learning

Unsupervised learning uses unlabeled input data to find the underlying structure and relationship in a dataset. It is a powerful tool for association and clustering tasks (Senders *et al.* 2018). The algorithms used in unsupervised learning include principal components analysis and clustering algorithms (Krittanawong *et al.* 2017, Camacho *et al.* 2018). Typical clustering algorithms are K-means (MacQueen 1967), hierarchical clustering (Johnson 1967) and spectral clustering (Ng *et al.* 2002). Since the correct output labels are unknown, the performance of clustering cannot be directly measured. Therefore, the performance depends on whether the clusters capture the trends in the data (Bastanlar & Ozuysal 2014).

Unsupervised learning is commonly used for deep learning and has been applied in autonomous vehicles, robots, speech recognition and pattern recognition (Krittanawong *et al.* 2017). In contrast to other methods, unsupervised learning and deep learning in health are still developing; their applications in the field of reproduction have been scarce so far (Miotto *et al.* 2018). Milewski *et al.* (2017) used PCA and ANN to create a model for implantation prediction based on the morphokinetic information recordings of 610 human embryos transferred in 514 cycles. The combination algorithm was efficient with the AUC as 75%. Li *et al.* (2014) proposed a method using PCA to extract image features and using the k-nearest neighbor (KNN) algorithm to diagnose human sperm health. Finally, the accuracy of healthy diagnosis was 95.73%, and the unhealthy diagnosis was 51.35%. The model based on PCA and KNN performed better than the others. However, the predicted results are still not ideal, and further research is needed. In addition to this, unsupervised clustering has been applied to the studies on sperm motility and morphometry subpopulations, which allow for the classification of sperm heterogeneity efficiently without any other prior information (Ramon & Martinez-Pastor 2018).

### Natural language processing

Natural language processing (NLP) is a subfield of AI that aims to understand human language and extract useful information from contents such as EMRs data to assist in clinical decision making (Hashimoto *et al.* 2018). The process of NLP includes text processing and classification (Jiang *et al.* 2017). NLP allows doctors to write more naturally, rather than having to record medical information within a specific framework to allow the computer to identify the data (Hashimoto *et al.* 2018). There are various applications of NLP in the medical domain, such as diagnostic surveillance and identifying medical cases for research studies (Tang *et al.* 2018). NLP has also been developed to monitor adverse effects. Miller *et al.* (2017) used NLP to automatically monitor laboratory adverse events. The main limitation is the generalizability of NLP systems, which requires local expert customization to accommodate specific nuances to improve its performance (Tang *et al.* 2018).

In the field of reproduction, big data means we need to research a vast amount of patients and integrate the various types of data. Using these types of data through AI holds great promise for identifying patterns that are beyond human understands. EMRs are typically used for recording and sharing medical information. The use of EMRs is a good solution for data collection in the research of reproductive medicine. EMRs digitally capture data on patient characteristics, treatment history, adverse events, physical examination, clinical laboratory reports and follow-up (Jiang *et al.* 2017). We can obtain enough data for subsequent machine learning process by extracting useful information from EMRs using NLP (see Fig. 2).

### Robotic surgery

Over the past three decades, the most influential innovation in surgery is the advent of minimally invasive surgery (MIS), which has changed the modern surgical practice by combining multiple technological developments, such as high-resolution cameras and micro-operated instruments, to enter the human body through small incisions for surgical procedures (Diana & Marescaux 2015). The minimally invasive approach is preferable to an open approach, especially for better perioperative outcomes, patient safety and quality of life, factors essential for optimizing patients’ reproductive outcomes. Recently, the application of robotic surgery provides surgeons with improved ergonomics, three-dimensional visualization, higher precision, fine instruments and shorter learning curves and overcomes the limitations of traditional laparoscopy (Lonnerfors 2018). Combining the superiority of AI and MIS, robotic surgery has been applied widely in many surgical fields and has shown advantages in complex gynecological diseases (Tan *et al.* 2012). Robotic technology applied to laparoscopy has been introduced into clinical practice and has enhanced the armamentarium of the reproductive specialist. Uterine leiomyomas, adenomyosis, endometriosis, adnexal masses, sterilization reversal and fertility preservation techniques can all benefit from robotic surgery (Estes *et al.* 2017). However, there are still little-randomized trials, and most studies published are observational or retrospective studies.

Robotic myomectomy has been used for more than 10 years and has achieved good safety and effectiveness. The study by Barakat *et al.* evaluates three methods for myomectomy, and the robot-assisted laparoscopic myomectomy has a decrease in blood loss, smaller scars, shorter duration of hospitalization and fewer postoperative complications (Advincula *et al.* 2007, Barakat *et al.* 2011). However, some studies (Nezhat *et al.* 2009, Gargiulo *et al.* 2012) have found a longer surgical time operating time with robotic myomectomy compared with standard laparoscopy; some studies (Bedient *et al.* 2009) observed no significant differences for surgical time, blood loss, complications, duration of hospitalization and readmissions. In general, robotic surgery in most studies is a viable alternative to abdominal uterine myomectomy and standard laparoscopic myomectomy with similar perioperative outcomes. Moreover, robotic surgery for deep-infiltrating endometriosis (DIE) has achieved comparable outcomes with conventional laparoscopy for treating endometriosis and improved reproductive outcomes (Nezhat *et al.* 2010, Brudie *et al.* 2012, Siesto *et al.* 2014, Magrina *et al.* 2015, Tan *et al.* 2018). For patients with adenomyosis, robotic surgery can offer the opportunity for meticulous suturing in multiple layers and 3D vision that can discern the boundaries between the myometrial lesions and healthy surrounding myometrium and has improved symptoms and resolution of adenomyosis (Chung *et al.* 2016). Robotic assistance allows us to perform this procedure in a minimally invasive approach; however, adenomyosis resection is still a surgical procedure with unclear clinical efficacy and unknown reproductive outcomes and there is a lack of studies of subsequent pregnancy outcomes for robotic adenomyomectomy. Furthermore, robotic surgery with a quicker learning curve and improved postural ergonomics has proven to be effective in tubal reanastomosis (Caillet *et al.* 2010, van Seeters *et al.* 2017), vasectomy reversal (Kavoussi 2015, Patel & Smith 2016, Marshall *et al.* 2017), female fertility preservation (Gargiulo 2011, Finger & Nezhat 2014, Oktay *et al.* 2016) and robotic ICSI (Lu *et al.* 2011).

The development and innovations of robotics and computer science have enhanced the surgeons’ skills to achieve accuracy and precision in complex surgeries. New minimally invasive approaches such as laparoendoscopic single‐site (LESS) surgery and natural‐orifice transluminal endoscopic surgery (NOTES) can reduce surgical trauma, operative complications and potentially improve outcomes. Due to poor ergonomics and a long-learning curve in traditional surgery, robotic surgery has good prospects. Studies have shown that single-site robotic surgery is feasible and safe in patients with gynecological disease (Scheib & Fader 2015, Bogliolo *et al.* 2016). NOTES procedures have been applied in cholecystectomies and appendectomies, and the risk of morbidity has not increased compared with traditional laparoscopic surgery (Jallad *et al.* 2016, Bulian *et al.* 2017). There are currently few studies using NOTES for gynecological surgery. Robotic surgery has a good clinical role in reproductive surgery. Randomized trials are needed to demonstrate the effectiveness of robotic surgery. With the further development of AI, we can continuously improve patient outcomes and the outcome of future fertility through robotic surgery.

## AI applications in reproductive medicine

### Evaluation and selection of oocytes

The overall success of reproduction, either spontaneously or after ART, is highly dependent upon the quality of oocytes. Currently, the pregnancy rate per retrieved oocyte is estimated at 4.5% (Stoop *et al.* 2012). A better understanding of the oocyte developmental competence will help guide the development of new strategies to improve the success rate of IVF and new biomarkers to predict oocyte quality and select the optimal egg for IVF (Conti & Franciosi 2018). A variety of strategies have been proposed to evaluate and select the oocyte with the best developmental potential, but kinds of limitations such as the possibility that the normal-appearing oocyte or embryo may still conceal aneuploidy (Munne *et al.* 2007, 2009) prompt more research to obtain precise standards and methods. Thus, the use of AI methods for oocyte selection in IVF programs may bring new opportunities. Cavalera *et al.* (2018) observed mouse oocytes during their *in vitro* maturation from the germinal vesicle (GV) to the metaphase II stage and took pictures for time-lapse analysis. They calculated the profile of cytoplasmic movement velocities by analyzing the images using the particle image velocimetry (PIV) method, and then the data were analyzed with a feed-forward artificial neural network to identify the competent or incompetent oocytes with an accuracy of 91.03%. Moreover, some researchers utilized noninvasive approaches to predict human oocyte developmental potential. Yanez *et al.* (2016) reported that viscoelastic properties of human zygotes measured nondestructively within hours after fertilization could reliably predict viability and blastocyst formation, with >90% precision, 95% specificity and 75% sensitivity. Furthermore, the researchers examined the RNA-seq data and found that non-viable embryos exhibited significantly different transcriptomes especially in the expression of genes important for oocyte maturation. The ideal method of oocyte selection would be noninvasive, inexpensive and capable of being incorporated into the embryology workflow with minimal impact (Kort & Behr 2017). ART still has room for improvement, such as the technologies for a more reliable prediction of oocyte quality and more accurate quantification of gamete developmental competence. Besides, applying AI methods to the evaluation of human oocytes that utilizes time-lapse (Faramarzi *et al.* 2017) or assesses gene expression through transcriptomics or genomics (Biase 2017, Freour & Vassena 2017) may have a good development prospect and further benefit ARTs.

### Sperm selection and semen analysis

Semen analysis is the first step in the evaluation of infertile couples. Sperm morphology reflects kinds of anomalies in human semen samples. The ability to identify the morphology of the sperm cells and to monitor the alterations in sperm motility is paramount to evaluating the potential fertility of a sample. Currently, the computer-aided sperm analysis (CASA) systems are used for research and routine analysis in human or animal. The system can report the motile percentage and kinematic parameters and identify the subpopulations of sperm cells (Goodson *et al.* 2017). Due to the inherent lack of objectivity and the difficulty in the manual evaluation of the sperm morphology and the high degree of variation between laboratories, the automatic methods based on image analysis should be developed to gain more objective and precise results. Besides, up to one-third of male factor infertility are idiopathic (Gudeloglu *et al.* 2014), which means that the current methods of assessing sperm cannot detect multiple causes of infertility. Goodson *et al.* (2011) developed an automated and quantitative method to classify the motility patterns of mouse sperm based on 2043 sperm tracks obtained from the CASA system. In 2017, (Goodson *et al.* 2017) applied the same method to human sperm. The overall accuracy of this model is 89.92%, retrospectively utilized the data of 425 human sperm to develop a model and diagnose chromosomal abnormalities. Height, total testicular volume, follicle-stimulating hormone, luteinizing hormone, total testosterone and ejaculate volume were used and the prediction of chromosomal abnormalities achieved more than 95% accuracy. Based on the data of the lifestyle and environmental features, data mining methods can also be used to make predictions for seminal quality. Girela *et al.* (2013) developed two specific neural networks to predict the human sperm concentration and motility based on environmental factors and lifestyle from questionnaires. Although the method seemed to be an alternative to more expensive laboratory tests, it could be a useful tool in early diagnosis. Sahoo & Kumar (2014) used five AI techniques to predict the fertility rate in human and applied eight feature selection methods to find out appropriate attributes that can predict the male fertility rate more accurately. The feature selection could improve performance, visualize the data for model selection, reduce dimensionality and remove noise efficiently. Finally, feature selection methods increased the accuracy of the AI techniques and support vector machine plus particle swarm optimization provided higher accuracy and AUC rate (94 and 0.932).

### Embryo selection

Precise assessment of embryo viability is a prime factor in maximizing pregnancy rate and optimizing of IVF treatments (Saeedi *et al.* 2017). In most cases, embryologists select the embryos or oocytes by a noninvasive examination based on visual observation focused on morphology and dynamic development during the blastocyst stage. The evaluation of embryos is subjective and thus are subject to inter- and intra-observer variation considering the existence of embryo scoring systems and the experience and expertise of the embryologists for the final success rate (Baxter Bendus *et al.* 2006, Santos Filho *et al.* 2012, Manna *et al.* 2013). Besides, the potential consequences of multiple pregnancy and the risks of serious complications such as pre-eclampsia and maternal hemorrhage are elevated as more than one embryo is transferred per cycle, despite the potential increase in success rate of embryo transfer (Bromer & Seli 2008).

The introduction of automatic morphological analyses of embryos or blastocysts in conjunction with AI is an attractive possibility. Santos Filho *et al.* (2012) proposed a method for image segmentation and classification of human blastocyst images with semi-automatic grading. They trained two SVM classifiers to grade the inner cell mass (ICM) and trophectoderm (TE) quality. By calculating the fractal dimension and mean thickness of TE and ICM image texture descriptors, the main morphological features of the blastocyst were well characterized. Furthermore, the adjustment of the microscope such as greater contrast and stronger boundaries of individual features may yield better image analysis. Singh *et al.* (2015) presented a novel algorithm in a fully automatic method for identifying TE region of human blastocysts. They utilized the Retinex algorithm to enhance the quality of the input images, eventually achieving an average shape accuracy of 87.8% to detect TE regions. Saeedi *et al.* (2017) introduced the first automatic method for joint segmentation of TE and ICM in human blastocyst images. Creating and testing a dataset of 211 blastocyst images of different grades, they reported accuracy of 86.6% for identification of TE and 91.3% for ICM. Embryo morphology remains the current tool for embryo selection for transfer. The data obtained from automatic image identification can provide an opportunity to objectively assess the embryos and analyze them in a more quantitative way.

Methods for embryo selection based on morphokinetic parameters have been published. Additionally, embryo assessment using the dynamic monitoring system (Time-Lapse (TL)) provides continuous information on the embryos’ developmental stage and morphokinetics, though the time-lapse algorithms remains questionable (Storr *et al.* 2018); some researchers do noy consider it as evidence of the benefits for embryo election (Kaser & Racowsky 2014, Armstrong *et al.* 2018). Carrasco *et al.* (2017) retrospectively analyzed 800 human embryos with known implantation data in an incubator with Time-Lapse system. They developed a model based on the analysis of morphokinetic parameters and the embryo morphology assessment on D3. The morphokinetics can exclude the embryos with the lowest implantation potential.

### The prediction of IVF outcome

Today, many couples suffering from infertility try to have a baby based on ART. However, due to the low clinical pregnancy rates and the high cost per cycle (De Geyter *et al.* 2018), many infertile families are under tremendous pressure. By constructing a functional IVF prediction model combined with AI, clinicians can tailor personalized treatment of subfertile couples and improve the pregnancy outcome of ART. Several papers have described models to predict IVF outcomes (Kaufmann *et al.* 1997, Jurisica *et al.* 1998, Venkat *et al.* 2004, Guh *et al.* 2011, Guvenir *et al.* 2015), where different AI methods have been used with the accuracies from 59 (Kaufmann *et al.* 1997) to 84.4% (Guvenir *et al.* 2015). Although the accuracy of predictions is gradually improving, there remain various problems and the model cannot be applied in clinical practice well. Further research is needed. In a study done by Hafiz *et al.* (2017), they utilized previous IVF/ICSI records to predict the outcome with an AUC of more than 80%. They found that the age of woman, number of the developed embryos and the serum estradiol level on the day of human chorionic gonadotropin administration were the optimal predictive features. The limitations of their work included the number of IVF/ICSI records and the missing values that decreased the accuracy of the classifiers. Even a methodologically excellent model is limited by the quality and magnitude of the input dataset from which it is trained. Efforts should be made to resolve these difficulties in subsequent research.

## Limitations and challenges of AI research

In the reproductive domain, a significant challenge lies in determining the best ways to implement AI in clinical work. Machine learning algorithms such as DT, SVMs and neural networks have been widely used and have achieved good results. However, state-of-the-art ML algorithms such as deep learning are still in the initial stage and have not been researched adequately. There are defects associated with the application of AI in clinical activities. ML models are mostly a black box, lacking a universal understanding of inner workings. This opacity has ethical and legal risks and liability issues, which may lead to distrust of patients and clinicians to AI. The institutions and the clinicians involved in the creation, validation and supervision of ML algorithms should be responsible for the outcomes (Kohli *et al.* 2017). It is also essential to have a detailed design and evaluation of ML models. The performance of ML is related to various factors, including the quantity and quality of the data. Small training datasets can lead to wrong decisions if they are biased in supervised learning (Krittanawong *et al.* 2017). ML algorithms, such as deep learning, require a significant amount of data for training; it may perform poorly if the data is insufficient. Selection bias from sample collection can result in poor performance of ML models in a clinical setting (Senders *et al.* 2018). It also emphasizes the importance of data collection and sharing, both of which will enable us to utilize the high-quality data efficiently.

Currently, AI research mainly focuses on image analysis of sperm cells and embryos and on outcome prediction of ART. The applications of early disease prediction and diagnosis, treatment and prognosis evaluation are relatively inadequate. More research is needed to promote the applications of AI in reproductive medicine. There are certain areas where AI technology has equaled or exceeded the performance of expert clinicians (Stewart *et al.* 2018), which raises a concern that the ML models may replace the doctor. We should believe that AI is just a tool to supplement and enhance the physician (Kohli *et al.* 2017). Machine learning can handle simple and repetitive tasks, saving doctors a lot of time and effort. Meanwhile, clinicians should not blindly follow the predictions of the ML models but should apply the results to clinical work. Clinicians should always consider whether the model is constructed reasonably and it is compatible with the actual clinical scenario (Senders *et al.* 2018).

## Future directions of AI in reproductive medicine

AI research has yielded tremendous benefits from the development of massive open datasets that provide high-quality training data. AI can help physicians select better sperm and embryos for ART accurately and efficiently, because physicians can process and interpret more data with greater depth than ever. Significant trends in big data analytics are expected to create high-quality evidence. Ongoing efforts to develop such datasets are likely to present enormous opportunities for further advances in reproductive medicine. Big data is the source of wisdom for the development of AI medicine, and data mining is the basic technology for this work. Collecting a large amount of valid data and analyzing and integrating them can pave the way for the subsequent applications of AI such as machine learning. AI can assist data mining in obtaining more high-quality data as well. We can collect data in various ways such as creating an interconnected network of patient data from across the world (Cahan & Cimino 2017). In addition, combining medical data from the EMRs, medical image, laboratory examinations, genetic information and health records with advanced AI methods can potentially change the way in which medicine is practiced.

The decision support system based on big data is a significant development direction in reproductive medicine. It can update in real-time through dynamic programming and reinforcement learning techniques, assisting doctors in making better clinical decisions based on patient clinical data. Learn, analyze and summarize medical information through NLP to build a large-scale dataset, and then utilize deep learning to learn these massive amounts of data to construct models. The models are continuously optimized by comparing expert diagnosis and apply in AI-assisted diagnosis eventually. Medical expert system based on AI-assisted diagnosis is the most representative and important application, which can assist doctors in solving complex medical problems and serve as an auxiliary tool for clinical practice. In healthcare, a well-known application of ML is IBM’s Watson Health (Gyawali 2018). It provides diagnosis and possible treatment options for cancer. The system can assist physicians in making decisions and predicting patient outcomes. The more information a patient offers, the greater the chance of an accurate output.

The IVF laboratory mechanization is also a significant prospect for development (Meseguer *et al.* 2012). Integrating the new technologies for the non-subjective sperm and embryo selection, oocyte denudation by mechanical removal of cumulus cells, oocyte positioning, fertilization, embryo culture and monitoring of embryo development into an automated device can effectively improve the efficiency and effect of ART. Therefore, the development of AI will benefit more infertility couples.

## Conclusion

In this paper, we outlined the basics of AI and machine learning, reviewed its applications in reproductive medicine, and discussed the limitations, challenges and future trends of AI. With the increasing availability of big data and the development of precision medicine, the applications of AI in the medical field will continue to grow. Despite various limitations, current AI technologies are well positioned to address well-defined issues in a variety of clinical domains. Such a system has the potential to improve pregnancy outcomes and patient care for infertility patients. Over time, we believe that the capabilities of AI techniques are likely to improve, and the integration of these solutions into practice can benefit patients and physicians by providing high-quality health care more effectively and accurately.

## Declaration of interest

The authors declare that there is no conflict of interest that could be perceived as prejudicing the impartiality of this review.

## Funding

This work was supported by the National Natural Science Foundation of China (81672085, 71871169, 81372804, 71373188, 30901586, U1333115); the Chinese Medical Association of Clinical Medicine special funds for scientific research projects (17020400709).
